# Smart solution for hard times: successful lithoplasty of an undilatable lesion

**DOI:** 10.1007/s12471-019-1261-2

**Published:** 2019-03-12

**Authors:** J. Vainer, A. Lux, M. Ilhan, R. A. L. J. Theunissen, S. Aydin, A. W. J. van ’t Hof

**Affiliations:** 1 Heart+Vascular Center, Maastricht UMC+, Maastricht, The Netherlands; 2 Faculty of Health, Medicine and Life Sciences, Maastricht University, Maastricht, The Netherlands; 3 VieCuri Medical Center, Venlo, The Netherlands

After unsuccessful percutaneous coronary interventions (PCI) with high-pressure balloons (40 atm) and rotational atherectomy (1.5 mm burr), a 70-year-old woman was re-admitted for lithoplasty-assisted PCI. Lithoplasty balloons (Shockwave Medical, Freemont, California) were developed based on the principles of kidney stone treatment. With an array of emitters they generate pulsatile, circumferential sonic pressure waves to selectively disrupt intimal and medial calcifications, usually resulting in calcium tears and focal dissections [1, 2].

**Fig. 1:**
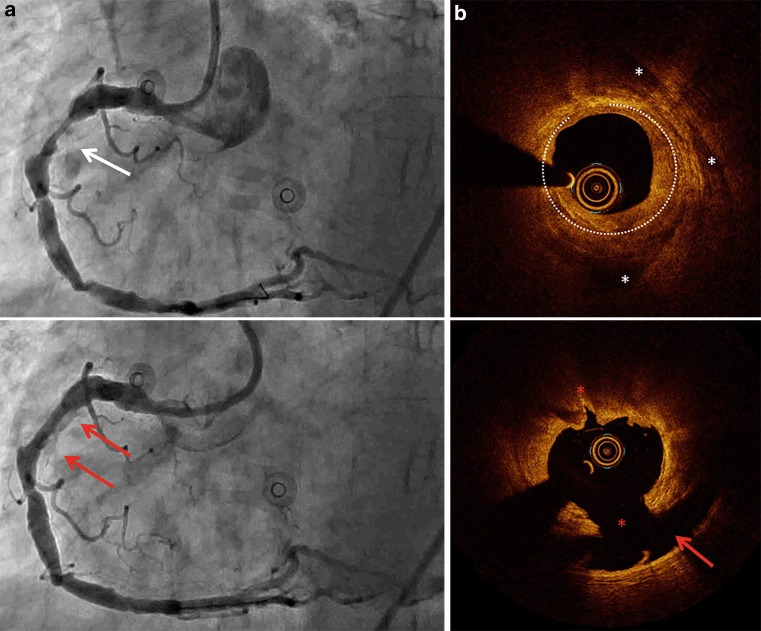
Images of the lesion before and after lithoplasty and OCT images. On the left (**a**) pre-lithoplasty (post-rotational atherectomy) and post-lithoplasty angiographic images of the diffusely diseased right coronary artery (RCA) are shown. Within the proximal segment a significant, extremely calcified lesion (*white arrow*) can be seen. Lithoplasty resulted in plaque modification and a significant increase in diameter. Residual contrast was noticed within the dissection flap (*red arrows*), and OCT images on the right (**b**) showed two tears (*red stars*) and an extensive dissection (*red arrow*) in the luminal ring and subintimal calcifications (*white dotted line and stars* respectively)

In this patient with Canadian Cardiovascular Society Class II angina, the 6 Fr compatible device effectively modified the extremely resistant lesion (Fig. 1a). Optical coherence tomography (OCT) showed typical calcium tears and a large dissection (Fig. 1b; [2–4]). To cover the lesion, a drug-eluting stent (4.5 mm) was implanted and post-dilated with a non-compliant balloon. Proper stent expansion and apposition were confirmed with OCT.

In conclusion, lithoplasty may become an essential and safe plaque modification tool, especially in coronary arteries with large inner diameters and subintimal calcifications [2].
